# Abnormal pulmonary hemodynamics during exercise is associated with exercise capacity in COPD

**DOI:** 10.1186/s12931-022-02238-9

**Published:** 2022-12-08

**Authors:** Teresa Sassmann, Philipp Douschan, Vasile Foris, Natascha Tröster, Katarina Zeder, Luka Brcic, Adrienn Tornyos, Gerhard Bachmaier, Michael Fuchsjäger, Horst Olschewski, Gabor Kovacs

**Affiliations:** 1 Division of Pulmonology, Department of Internal Medicine, Medical University of Graz, Graz, Austria; 2 Ludwig Boltzmann Institute for Lung Vascular Research, Stiftingtalstrasse 24, 8010 Graz, Austria; 3 Diagnostic and Research Institute of Pathology, Medical University of Graz, Graz, Austria; 4 Division of General Radiology, Department of Radiology, Medical University of Graz, Graz, Austria; 5 Institute for Medical Informatics, Statistics and Documentation, Medical University of Graz, Graz, Austria

**Keywords:** Exercise hemodynamics, Exercise capacity, Right heart catheterization, COPD

## Abstract

**Background:**

Pulmonary hypertension (PH) is a frequent complication in COPD and it is associated with decreased exercise capacity and poor prognosis. We hypothesized that even in COPD patients without significant PH at rest, abnormal pulmonary hemodynamics during exercise affect exercise capacity.

**Methods:**

Consecutive COPD patients with clinically indicated right heart catheterization and resting mean pulmonary arterial pressure (mPAP) < 25 mmHg and age- and sex-matched controls with the same limits of pulmonary hemodynamics but no chronic lung disease who underwent clinical work-up including invasive hemodynamic assessment during exercise, were retrospectively analyzed. Chi-square tests were used to evaluate differences between groups for categorical data and Fisher’s exact test or Mann–Whitney-U-tests for continuous variables. Associations were analyzed with Spearman rank correlation tests.

**Results:**

We included n = 26 COPD patients (female/male: 16/10, 66 ± 11 yr, FEV_1_: 56 ± 25%predicted) and n = 26 matched controls (FEV_1_: 96 ± 22%predicted). At rest, COPD patients presented with slightly increased mPAP (21 (18–23) vs. 17 (14–20) mmHg, p = 0.022), and pulmonary vascular resistance (PVR) [2.5 (1.9–3.0) vs. 1.9 (1.5–2.4) WU, p = 0.020] as compared to controls. During exercise, COPD patients reached significantly higher mPAP [47 (40–52) vs. 38 (32–44) mmHg, p = 0.015] and PVR [3.1 (2.2–3.7) vs. 1.7 (1.1–2.9) WU, p = 0.028] values despite lower peak exercise level [50 (50–75) vs. 100 (75–125) Watt, p = 0.002]. The mPAP/cardiac output slope was increased in COPD vs. controls [6.9 (5.5–10.9) vs. 3.7 (2.4–7.4) mmHg/L/min, p = 0.007] and negatively correlated with both peak oxygen uptake (r = − 0.46, p = 0.007) and 6-min walk distance (r = − 0.46, p = 0.001).

**Conclusion:**

Even in the absence of significant PH at rest, COPD patients reveal characteristic abnormalities in pulmonary hemodynamics during exercise, which may represent an important exercise-limiting factor.

**Supplementary Information:**

The online version contains supplementary material available at 10.1186/s12931-022-02238-9.

## Introduction

Airway obstruction and loss of functional pulmonary parenchyma are important features of chronic obstructive pulmonary disease (COPD), leading to impaired exercise capacity and survival [1]. In addition, pulmonary hypertension (PH) may complicate the course of COPD and contribute to poor prognosis [2–5]. The overall prevalence of a mean PAP ≥ 25 mmHg in COPD is estimated to be around 10% [6]. Although severe PH is rare, mild to moderate forms of pulmonary vascular disease (PVD) are quite frequent [7, 8]. Despite normal or mildly elevated pulmonary arterial pressures at rest, hemodynamic response to exercise may be abnormal in these patients, which could substantially contribute to their symptoms and impaired exercise capacity [5, 9, 10]. In recent years, our understanding of pulmonary hemodynamics during exercise improved and prognostically relevant variables have been identified. Out of these, the mPAP/CO-slope may be considered as most robust parameter to characterize abnormal pulmonary hemodynamics during exercise [11–13]. However, there are still very limited data available on the characteristic changes in pulmonary hemodynamics during exercise in COPD and on the clinical relevance of these changes.

In this study, we aimed to investigate pulmonary hemodynamics and right ventricular function during exercise in COPD patients with normal or slightly elevated mean pulmonary arterial pressure (mPAP) at rest and to compare them to age- and sex-matched controls. We hypothesized that patients with COPD may show an abnormal pulmonary hemodynamic response to exercise and that this is associated with reduced exercise capacity.

## Methods

### Study design, patients and ethics

All consecutive COPD subjects who underwent clinically indicated right heart catheterization (RHC) for suspected PH at our clinic between 2005 and 2018 were carefully clinically evaluated and the complete dataset resulting from these investigations was included into a local database, the GRAz Pulmonary Hypertension In COPD (GRAPHIC) registry [2]. For this retrospective analysis, all patients with resting mPAP ≥ 25 mmHg who underwent an exercise test with indwelling catheter, were included. The indication for resting RHC followed international guidelines [14]. Patients who turned out to have resting mPAP < 25 mmHg at RHC underwent exercise-RHC to gain additional information regarding mechanisms of dyspnea and exercise limitation. During their clinical work-up, patients underwent pulmonary function test, transthoracic echocardiography, six-minute-walk distance (6MWD), cardiopulmonary exercise testing (CPET), blood-gas analysis and laboratory testing. The diagnosis of COPD and the severity of airflow limitation were established according to the GOLD recommendations [15] by two independent respiratory physicians. If more than one RHC was performed during the observation period, we included only the first investigation.

The control group consisted of age- and sex-matched patients who were admitted to our clinic due to suspected PH or unexplained dyspnea during the same time period, but had no chronic lung disease, no obstructive or restrictive changes (FEV1/FVC > 70%, TLC > 80%), a peak oxygen uptake (VO_2_) ≥ 80% predicted and a mPAP < 25 mmHg and pulmonary arterial wedge pressure (PAWP) ≤ 15 mmHg at RHC at rest. Controls underwent the same diagnostic procedures as the COPD patients. The study protocol conformed to the Declaration of Helsinki and was approved by the Ethics Committee of the Medical University of Graz (EK 32–352 ex 19–20).

### Assessment of resting and exercise hemodynamics

Patients underwent routine resting RHC in the supine position using a Swan-Ganz catheter as previously described [16]. The Swan-Ganz catheter was percutaneously inserted via a jugular vein under local anesthesia. The zero reference level was set at the mid-thoracic level [17, 18]. Symptom-limited exercise RHC was performed on a cycle-ergometer with stepwise (25 Watt) increasing workloads every 2 min. Pulmonary pressure readings of systolic, diastolic and mean pulmonary arterial pressure (sPAP, dPAP, mPAP), PAWP and right atrial pressure (RAP) were performed at each exercise level and were averaged over a minimum of three respiratory cycles. Cardiac output (CO) was determined by thermodilution. Blood gas analysis was performed at rest and during peak exercise by an ABL-800-Flex blood gas analyzer (Drott®). Pulmonary vascular resistance (PVR) was calculated as (mPAP-PAWP)/CO, total pulmonary resistance (TPR) was determined as mPAP/CO and transpulmonary pressure gradient (TPG) as mPAP-PAWP. Pressure/flow slopes were calculated from the differences between peak exercise and resting mPAP (or PAWP, RAP or TPG) and the differences between peak exercise and resting CO [12, 19]. Pulmonary vascular compliance (PVC) was calculated by stroke volume (SV)/(sPAP–dPAP) and pulmonary artery (PA) stiffness index by 1/PVC per body surface area (BSA) [20, 21]. Of note, the pulmonary vascular compliance (PVC) represents a measure of pulsatile afterload of the right ventricle. PVC has been shown to be of prognostic relevance in PH and decreased values may help to identify early forms of pulmonary vascular disease [20, 22–26]. Pulmonary artery stiffness is related to PH and COPD severity and correlates with mPAP and PVR during exercise [27, 28].

For assessment of right ventricular function during exercise, we calculated the right ventricular output ratio RVOR = CI_max-rest_/CI_rest_, which shows the ability of the subject to increase cardiac output during exercise and is associated with prognosis in pulmonary hypertension patients [29–31].

### Statistical analysis

Normally distributed variables were represented as mean ± SD and not normally distributed variables as median and interquartile range for continuous data. Categorical data are presented as absolute and relative numbers and between the groups compared using chi-square tests. Differences between the groups in continuous variables were analyzed using Mann–Whitney-U-test for nonparametric variables. Associations were analyzed with Spearman rank correlation coefficient. Significance level was defined as p-value < 0.05. For statistical analysis, IBM SPSS Statistics 26 was used.

## Results

### Clinical and demographic characteristics

**Fig. 1:**
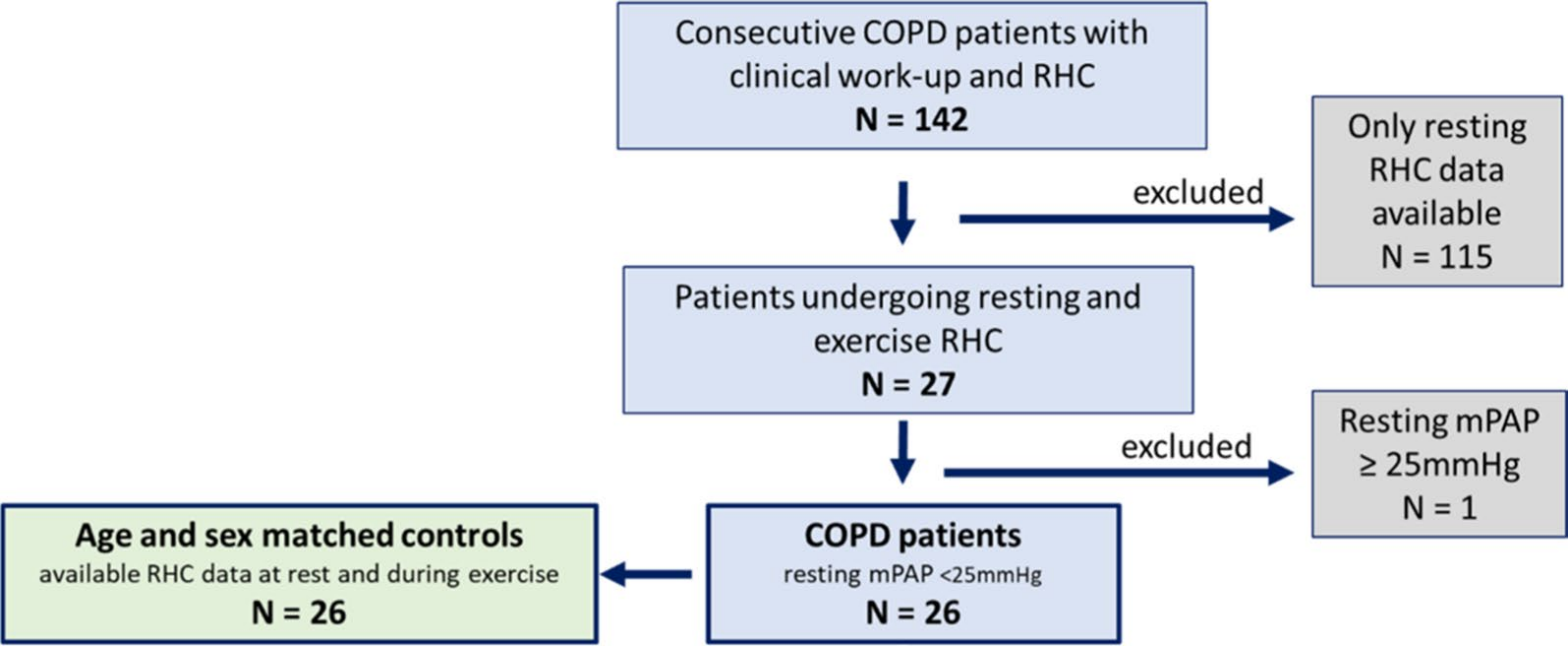
Flow chart of the cohort

A total of 142 COPD patients who underwent RHC between 2005 and 2018 were identified in our database. Out of these, in n = 115, only resting RHC was performed and in 27 subjects, an additional symptom-limited exercise RHC was performed. One patient was excluded because resting mPAP was ≥ 25 mmHg (29 mmHg) at rest. The remaining 26 patients were matched according to age- and sexto controls (Fig. 1). Table 1 shows the baseline characteristics of the patients. There were no significant clinical differences between the two groups, except for lung function parameters and 6MWD. For comorbidities see Additional file 1: Table S1.

**Table 1:** Patient characteristics

Variable	COPD N = 26	Controls N = 26	p-value
Age, yr	66 ± 11	66 ± 10	p = 0.990
Sex, (male/female)	10/16	10/16	–
	38%/62%	38%/62%	
BMI, kg/m^2^	24 ± 5	26 ± 4	p = 0.060
Systolic blood pressure, mmHg	126 ± 19	129 ± 18	p = 0.458
Diastolic blood pressure, mmHg	66 ± 11	68 ± 12	p = 0.469
WHO-FC			
I/II/III/IV	1/16/7/2	4/16/6/0	p = 0.098
	4%/62%/27%/8%	15%/62%/23%/0%	
Gold-stage			
I/II/III/IV	5/13/3/5	–	–
	19%/50%/12%/19%		
Smoking status			
Never/quit/active	7/15/4	12/12/2	p = 0.314
Pack years (py)	25 (11–33)	15 (9–30)	p = 0.323
LTOT treatment, n	5	0	–
Hb, g/dL	13.6 (12.7–14.9)	14.0 (12.6–14.7)	p = 0.880
NT-proBNP, pg/mL	263 (101–625)	144 (64–344)	p = 0.109
Creatinine, mg/dL	0.91 (0.70–1.28)	0.97 (0.81–1.07)	p = 0.497
FVC, % predicted	74 ± 25	101 ± 23	p = 0.001
FEV1, % predicted	56 ± 25	96 ± 22	p < 0.001
FEV1/FVC	60 (50–66)	78 (73–82)	p < 0.001
TLC, % predicted	123 ± 31	104 ± 12	p = 0.025
DLCO_c_SB, % predicted	59 ± 19	83 ± 18	p < 0.001
DLCO_c_VA, % predicted	74 ± 22	89 ± 17	p = 0.007
6MWD, m	360 (308–434)	447 (394–493)	p = 0.018
Maximal exercise level, Watt	50 (50–75)	100 (75–125)	p = 0.002
Peak VO_2_, %predicted	64 (55–82)	95 (87–102)	p < 0.001
E/E′ ratio	9 ± 2	12 ± 4	p = 0.140
LVEF, %	70 ± 8	67 ± 6	p = 0.465
TRV, mmHg	37 (31–42)	34 (31–40)	p = 0.808
sPAP, mmHg	42 (36–47)	41 (36–46)	p = 0.674
TAPSE, mm	24 (18–27)	25 (21–27)	p = 0.419

Normally distributed values are expressed as mean ± SD; non-parametric variables are expressed as median and interquartile range

*BMI* Body mass index, *WHO-FC* world health organization functional class, *Hb* hemoglobin, *NT-proBNP* N-terminal pro-brain natriuretic peptide, *FVC* forced vital capacity, *FEV1* forced expiratory volume in the first record of expiration, *TLC* total lung capacity, *DLCOc* diffusing capacity of lung for carbon monoxide, *SB* single-breath, *DLCOcVA* diffusing capacity of lung for carbon monoxide for alveolar volume corrected for hemoglobin, *TAPSE* tricuspid annular plane systolic excursion, *VO*_*2*_ oxygen uptake, *E/E’* ratio of transmitral early filling velocity to early diastolic tissue velocity, *LVEF* left ventricular ejection fraction, *TRV* tricuspid regurgitation velocity, *sPAP* systolic pulmonary arterial pressure

### Pulmonary hemodynamics at rest

The hemodynamic data derived from resting RHC are provided in Table 2. Compared to the control group, COPD patients had slightly elevated mPAP [21 (18–23) mmHg vs. 17 (14–20) mmHg, p = 0.022], PVR [2.5 (1.9–3.0) WU vs. 1.9 (1.5–2.4) WU, p = 0.035] and TPR [4.3 (3.8–5.7) WU vs. 3.5 (2.7–4.4) WU, p = 0.007], while there were no significant differences in PAWP, RAP and CO as well as in pulmonary arterial compliance and stiffness. Out of the resting hemodynamic parameters only mPAP and TPR were significantly associated with peak VO_2_ and none of the assessed parameters was associated with 6MWD.

**Table 2:** Pulmonary hemodynamics and right ventricular function at rest and during exercise

Variable	COPD N = 26	Controls N = 26	p-value
RHC at rest			
mPAP, mmHg	21 (18–23)	17 (14–20)	p = 0.022
PAWP, mmHg	8 (6–10)	8 (6–10)	p = 0.789
RAP, mmHg	5 (3–6)	5 (4–6)	p = 0.492
PVR, WU	2.5 (1.9–3.0)	1.9 (1.5–2.4)	p = 0.020
TPR, WU	4.3 (3.8–5.7)	3.5 (2.7–4.4)	p = 0.007
CO, L/min	4.4 (3.8–5.8)	4.5 (4.0–5.3)	p = 0.742
CI, L/min/m^2^	2.6 (2.3–3.1)	2.6 (2.3–2.8)	p = 0.782
PVC, mL/mmHg	3.7 (2.7–5.0)	4.0 (3.0–5.1)	p = 0.351
PA stiffness index, mmHg/m^2^/mL	0.50 (0.37–0.62)	0.45 (0.35–0.59)	p = 0.498
RHC at 25 Watt			
mPAP, mmHg	36 (30–41)	29 (21–35)	p = 0.025
PAWP, mmHg	13 (11–23)	13 (11–17)	p = 0.302
RAP, mmHg	8 (6–13)	7 (4–11)	p = 0.271
PVR, WU	3.0 (1.9–4.0)	2.2 (1.2–2.6)	p = 0.110
TPR, WU	5.2 (4.1–7.3)	3.9 (3.1–5.1)	p = 0.024
CO, L/min	6.6 (5.3–8.5)	7.0 (5.8–8.4)	p = 0.323
CI, L/min/m^2^	3.7 (3.2–4.7)	3.8 (3.4–4.6)	p = 0.534
PVC, mL/mmHg	4.3 (2.4–5.5)	3.6 (2.5–6.2)	p = 0.607
PA stiffness index, mmHg/m^2^/mL	0.49 (0.32–0.82)	0.50 (0.41–0.77)	p = 0.442
RHC at 50 Watt			
mPAP, mmHg	46 (39–51)	31 (24–41)	p = 0.006
PAWP, mmHg	19 (12–26)	16 (12–21)	p = 0.203
RAP, mmHg	12 (9–17)	9 (5–13)	p = 0.068
PVR, WU	2.8 (1.9–3.5)	1.8 (1.1–2.5)	p = 0.053
TPR, WU	5.4 (4.2–6.6)	3.7 (2.7–4.8)	p = 0.013
CO, L/min	8.1 (7.1–9.7)	8.6 (7.8–10.0)	p = 0.273
CI, L/min/m^2^	4.6 (4.3–5.5)	4.9 (4.4–5.3)	p = 0.396
PVC, mL/mmHg	2.0 (1.7–2.5)	3.6 (2.4–4.5)	p = 0.010
PA stiffness index, mmHg/m^2^/mL	0.84 (0.72–1.05)	0.53 (0.41–0.77)	p = 0.023
RHC at individual peak exercise			
Maximal exercise level, Watt	50 (50–75)	100 (75–125)	p = 0.002
Peak VO_2_, %predicted	64 (55–82)	95 (87–102)	p < 0.001
mPAP, mmHg	47 (40–52)	38 (32–44)	p = 0.015
PAWP, mmHg	20 (15–28)	20 (15–25)	p = 0.495
RAP, mmHg	14 (8–18)	11 (7–14)	p = 0.165
PVR, WU	3.1 (2.2–3.7)	1.7 (1.1–2.9)	p = 0.028
TPR, WU	5.7 (4.5–7-0)	3.8 (2.6–5.2)	p = 0.005
CO, L/min	7.8 (7.0–10.9)	10.3 (8.1–13.5)	p = 0.044
CI, L/min/m^2^	4.7 (4.2–5.7)	6.2 (4.5–7.9)	p = 0.034
PVC, mL/mmHg	1.9 (1.6–2.6)	3.0 (2.0–4.4)	p = 0.045
PA stiffness index, mmHg/m^2^/mL	0.84 (0.70–1.11)	0.61 (0.42–1.00)	p = 0.056
RVOR (CI_exercise-rest_/CI_rest_)	0.9 (0.5–1.2)	1.3 (0.7–1.8)	p = 0.020
Slopes			
mPAP/CO slope, mmHg/L/min	6.9 (5.0–10.9)	3.7 (2.4–7.4)	p = 0.007
PAWP/CO slope, mmHg/L/min	2.9 (1.9–6.4)	1.8 (1.3–4.2)	p = 0.051
RAP/CO slope, mmHg/L/min	2.3 (1.3–4.2)	1.2 (0.5–2.1)	p = 0.025
TPG/CO slope, mmHg/L/min	3.5 (1.6–4.5)	1.6 (0.9–3.5)	p = 0.048

Normally distributed values are expressed as mean ± SD; non-parametric variables are expressed as median and interquartile range. In n = 8 COPD and n = 2 control patients maximal exercise level was 25 W

*6MWD* six-minute walk test, *VO*_*2*_ oxygen uptake, *mPAP* mean pulmonary artery pressure, *PAWP* pulmonary artery wedge pressure, *RAP* right atrial pressure, *PVR* pulmonary vascular resistance, *TPR* total pulmonary resistance, *CI* cardiac index, *CO* cardiac output, *PVC* pulmonary vascular compliance, *PA stiffness* pulmonary artery stiffness, *RVOR* right ventricular output reserve

### Pulmonary hemodynamics during exercise

**Fig. 2:**
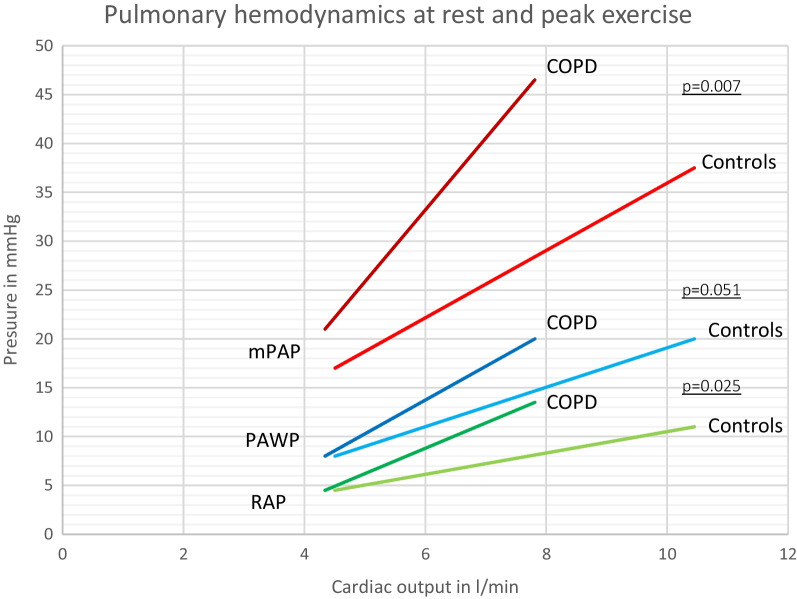
MPAP/CO slope; PAWP/CO slope and RAP/CO slope in COPD and controls. *mPAP* mean pulmonary artery pressure, *PAWP* pulmonary artery wedge pressure, *RAP* right atrial pressure, *CO* cardiac output

During exercise, COPD patients, as compared to controls, reached significantly lower maximal exercise levels [50 (50–75) Watt vs. 100 (75–125) Watt, p = 0.002], and had impaired peak oxygen uptake [peak VO_2_ 64 (48–80) %predicted vs. 97 (87–102) %predicted, p < 0.001] and 6MWD [360 (289–441) m vs. 449 (329–493) m, p = 0.033)]. Resting hemodynamic differences between COPD and controls became more pronounced during exercise (Table 2). COPD patients had steeper mPAP/CO [6.9 (5.0–10.9) mmHg/L/min vs. 3.7 (2.4–7.4) mmHg/L/min, p = 0.007], RAP/CO [2.3 (1.3–4.2) mmHg/L/min vs. 1.2 (0.5–2.1) mmHg/L/min, p = 0.025], TPG/CO [3.5 (1.6–4.5) mmHg/L/min vs. 1.6 (0.9–3.5) mmHg/L/min, p = 0.048] and PAWP/CO-slopes [COPD: 2.9 (1.9–6.4) vs. controls: 1.8 (1.3–4.2) mmHg, p = 0.051] as compared to controls (Fig. 2). RVOR was significantly decreased in COPD patients as compared to controls [0.9 (0.5–1.2) vs. 1.3 (0.7–1.8), p = 0.020].

### Association between exercise capacity and pulmonary hemodynamics

**Fig. 3:**
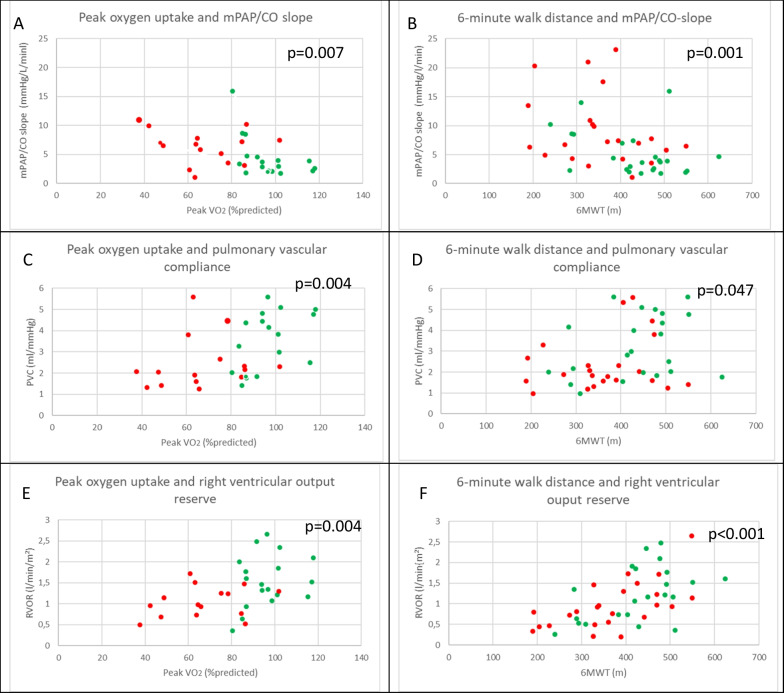
The relationship between cardiopulmonary exercise testing derived peak oxygen uptake (Peak VO_2_) and 6-min walk test (6MWT) with mean pulmonary arterial pressure/cardiac output slope (mPAP/CO slope (**A**, **B**), pulmonary vascular compliance (PVC) (**C**, **D**) and right ventricular output reserve (RVOR) (**E**, **F**). Red dots represent COPD patients, green dots controls

Several parameters of exercise hemodynamics were associated with peak VO_2_ and 6MWD (Table 3 and Fig. 3). The mPAP/CO slope, PVC at peak exercise, and RVOR showed the strongest correlations with both peak VO_2_ and 6MWD.

**Table 3:** Correlation of exercise capacity with pulmonary hemodynamics, right ventricular function and pressure/cardiac output slopes of all subjects

Value	Peak oxygen uptake (% predicted)		6 min walk distance (m)	
	Spearman correlation	p-value	Spearman correlation	p-value
Resting meanPAP, mmHg	− 0.461	p = 0.007	− 0.145	p = 0.338
Resting PAWP, mmHg	− 0.126	p = 0.485	0.071	p = 0.639
Resting RAP, mmHg	0.042	p = 0.817	0.015	p = 0.920
Resting CI, L/min/m^2^	− 0.222	p = 0.241	− 0.105	p = 0.487
Resting TPR, WU	− 0.389	p = 0.025	− 0.249	p = 0.095
Resting PVR, WU	− 0.318	p = 0.072	− 0.239	p = 0.109
Peak meanPAP, mmHg	− 0.445	p = 0.009	− 0.019	p = 0.901
Peak PAWP, mmHg	0.052	p = 0.776	− 0.204	p = 0.184
Peak RAP, mmHg	− 0.264	p = 0.138	0.116	p = 0.441
Peak CI, L/min/m^2^	0.337	p = 0.055	0.542	p < 0.001
Peak TPR, WU	− 0.422	p = 0.016	− 0.432	p = 0.003
Peak PVR, WU	− 0.517	p = 0.003	− 0.155	p = 0.333
Peak PVC, mL/mmHg	0.490	p = 0.004	0.301	p = 0.047
Peak PA stiffness index, mmHg/m^2^/mL	− 0.562	p = 0.001	− 0.260	p = 0.089
RVOR, L/min/m^2^	0.492	p = 0.004	0.577	p < 0.001
mPAP/CO slope, mmHg/L/min	− 0.459	p = 0.007	− 0.464	p = 0.001
PAWP/CO slope, mmHg/L/min	− 0.189	p = 0.293	− 0.547	p < 0.001
RAP/CO slope, mmHg/L/min	− 0.331	p = 0.064	− 0.215	p = 0.155
TPG/CO slope, mmHg/L/min	− 0.423	p = 0.014	− 0.173	p = 0.250

*mPAP* mean pulmonary artery pressure, *PAWP* pulmonary artery wedge pressure, *RAP* right atrial pressure, *PVR* pulmonary vascular resistance, *CO* cardiac output, *PVC* pulmonary vascular compliance, *PA stiffness* pulmonary artery stiffness, *RVOR* right ventricular output reserve, *TPG* transpulmonary gradient

## Discussion

In this study, we show that COPD patients, even without significant pulmonary hypertension at rest, present with characteristic changes in pulmonary hemodynamics and right ventricular function during exercise as compared to age- and sex-matched controls without chronic lung disease. The significant association between these changes and exercise capacity highlights the clinical relevance of these findings.

### Pulmonary hemodynamics at rest and exercise in COPD

According to the available studies, elevation of resting mPAP in COPD patients is associated with clinical worsening and progression to PH [32, 33], however, the clinical relevance of exercise hemodynamics, which may help to reveal the pathophysiology of exercise dyspnea, has only rarely been investigated in these patients. A recent study, providing new insights into specific characteristics of exercise pulmonary hemodynamics in COPD, described a strong increase in mPAP on effort [10]. In addition, a small previous study in COPD patients undergoing lung volume reduction surgery, revealed that the wall thickness of pulmonary vessels was not related to mPAP at rest, but was strongly correlated with mPAP during exercise (r = 0.721, p = 0.02) and with the change in mPAP from rest to exercise (r = 0.899, p = 0.0004) [34]. Our results complement these findings, because we provide a characterization of pulmonary hemodynamics during exercise in COPD and show significant associations between changes in exercise hemodynamics and decreased exercise capacity, suggesting potential mechanisms of exercise limitation in these patients.

### Pulmonary pressure-cardiac output slopes

There is growing evidence for the importance of mPAP/CO slope in exercise testing for predicting clinical outcomes [11, 13]. According to recent studies, healthy subjects have mPAP/CO slopes of 1.6 to 3.3 mmHg/L/min, whereas patients with PVD typically show values > 3 mmHg/L/min [11, 13]. A mPAP/CO slope > 3 mmHg/L/min was independently associated with hospitalization and survival in patients with chronic exertional dyspnea and preserved ejection fraction and a mPAP/CO slope ≥ 2.9 mmHg/L/min was significantly correlated with age-adjusted mortality in systemic sclerosis [13, 35]. Moreover, the predictive value of this slope is independent from pulmonary resting hemodynamics [36]. The mPAP/CO slope of our COPD cohort was increased to 6.9 mmHg/L/min, indicating a strongly abnormal pulmonary hemodynamic response to exercise. In addition, peak VO_2_ was significantly negatively associated with the mPAP/CO-slope, suggesting that pathological exercise hemodynamics may significantly contribute to exercise limitation. This might explain the exercise induced dyspnea of these patients. In order to further explore the underlying reason of mPAP elevation at exercise, we assessed the changes in TPG, PAWP and RAP during exercise. We found that all pressure/CO slopes were steeper in COPD patients as compared to controls, suggesting that both pre- and postcapillary factors and an additional increase in intrathoracic pressure may significantly contribute to abnormal pulmonary hemodynamics during exercise in COPD.

### Impact of right ventricular function

Apart from the involvement of pulmonary vessels, also changes in right ventricular function and its adaptation to increased afterload may be of prognostic importance and even early signs of RV failure may be associated with poor outcome [31, 37–40]. RVOR, among other factors, depends on the ability of the right ventricle to increase cardiac output during exercise. RVOR was significantly reduced in COPD patients as compared to controls (0.9 L/min/m^2^ vs. 1.3 L/min/m^2^, p = 0.02), which might suggest a limited right ventricular reserve in COPD patients. In addition, RVOR was significantly associated with peak VO_2_ and 6MWD, suggesting an important contribution to exercise limitation in COPD. Of note, RV function at rest as characterized by echocardiography (tricuspid annular plane systolic excursion), did not differ between COPD patients and controls.

### Limitations

The retrospective nature and the relatively low number of included subjects are obvious limitations of the current study. This limitation may at least partly be compensated by the thorough invasive hemodynamic characterization by an experienced clinical team. The relatively small number of included patients did not allow to perform multivariate analysis and to investigate mortality as an endpoint. Nevertheless, the clear associations with exercise capacity underline the clinical relevance of pulmonary hemodynamics during exercise in COPD. Another limitation is the absence of a truly healthy control group which was not available due to ethical reasons along with the invasive nature of the investigation.

## Conclusions

Even in the absence of more than mild pulmonary hypertension, COPD patients show characteristic abnormalities in pulmonary hemodynamics and right ventricular function during exercise, which may significantly contribute to their exercise limitation.

## Supplementary Information

**Additional file 1: Table S1.** relevant cardiac, pulmonary, hepatic and renal comorbidities from both groups are listed. “Minor intracardiac shunt” was one atrial septum defect without indication for intervention (COPD), one status post occlusion of a foramen ovale 10 years before (control). **Table S2.** Follow-up right heart catheterization (RHC) and all-cause mortality in COPD patients and controls.

## Data Availability

Not applicable.

## Abbreviations

6MWD: 6-Minute walk distance
BMI: Body mass index
BSA: Body surface area
CI: Cardiac index
CO: Cardiac output
COPD: Chronic obstructive pulmonary disease
CPET: Cardiopulmonary exercise testing
DLCO: Diffusing capacity of lung for carbon monoxide
DLCOcVA: Diffusing capacity of lung for carbon monoxide for alveolar volume corrected for hemoglobin
dPAP: Diastolic pulmonary arterial pressure
FEV_1_: Forced expiratory volume in the first record of expiration
FVC: Forced vital capacity
Hb: Hemoglobin
mPAP: Mean pulmonary arterial pressure
NtproBNP: N-terminal pro-brain natriuretic peptide
PA: Pulmonary artery
PAWP: Pulmonary arterial wedge pressure
PH: Pulmonary hypertension
PVC: Pulmonary vascular compliance
PVR: Pulmonary vascular resistance
RAP: Right atrial pressure
RHC: Right heart catheterization
RVOR: Right ventricular output ratio
sPAP: Systolic pulmonary arterial pressure
SV: Stroke volume
TAPSE: Tricuspid annular plane systolic excursion
TLC: Total lung capacity
TPG: Transpulmonary pressure gradient
TPR: Total pulmonary resistance
VO2: Oxygen uptake
WHO-FC: World health organization functional class
WU: Wood units
