# Supplementary material for: Abnormal pulmonary hemodynamics during exercise is associated with exercise capacity in COPD
Source: Respir Res. 2022 Dec 8;23:331. doi: 10.1186/s12931-022-02238-9 (PMC9733173; doi:10.1186/s12931-022-02238-9)
Supplement: Supplementary file 1 — Additional file 1: Table S1. relevant cardiac, pulmonary, hepatic and renal comorbidities from both groups are listed. “Minor intracardiac shunt” was one atrial septum defect without indication for intervention (COPD), one status post occlusion of a foramen ovale 10 years before (control). Table S2. Follow-up right heart catheterization (RHC) and all-cause mortality in COPD patients and controls. [file 12931_2022_2238_MOESM1_ESM.docx]

**Additional file 1**

| Comorbidities | | COPD  N=26 | Controls  N=26 |
| --- | --- | --- | --- |
| cardiac | Systemic arterial hypertension | 10 | 10 |
|  | Atrial Fibrillation | 5 | 6 |
|  | Coronary artery disease | 1 | 6 |
|  | Minor intracardiac shunt | 1 | 1 |
| pulmonary | Asthma bronchiale, controlled | - | 1 |
|  | Obstructive sleep apnea/ noninvasive ventilation therapy | 4/1 | 1/0 |
|  | Systemic sclerosis with mild pulmonary involvement | 2 | 2 |
|  | Chronic thromboembolic disease | 0 | 3 |
| hepatic | Liver cirrhosis Child A/B/C | 1/0/0 | 0/2/0 |
| renal | Severely decreased kidney function (GFR <30 ml/min) | 1 | 2 |

*Table S1 relevant cardiac, pulmonary, hepatic and renal comorbidities from both groups are listed. “Minor intracardiac shunt” was one atrial septum defect without indication for intervention (COPD), one status post occlusion of a foramen ovale 10 years before (control)*

|  | COPD N=26 | Controls N=26 |
| --- | --- | --- |
| Follow-up RHC | 6 | 6 |
| Development of mPAP≥25mmHg/ Initiation of PAH Therapy | 1/1 | 1/0 |
| All-cause mortality during follow-up | 3 | 5 |

*Table S2 Follow-up right heart catheterization (RHC) and all-cause mortality in COPD patients and controls.*
